# Gender-sensitive reporting in medical research

**DOI:** 10.1186/1758-2652-15-11

**Published:** 2012-03-08

**Authors:** Shirin Heidari, Quarraisha Abdool Karim, Judith D Auerbach, Simone E Buitendijk, Pedro Cahn, Mirjam J Curno, Catherine Hankins, Elly Katabira, Susan Kippax, Richard Marlink, Joan Marsh, Ana Marusic, Heidi M Nass, Julio Montaner, Elizabeth Pollitzer, Maria Teresa Ruiz-Cantero, Lorraine Sherr, Papa Salif Sow, Kathleen Squires, Mark A Wainberg

**Affiliations:** 1 International AIDS Society, Geneva, Switzerland; 2 Centre for the AIDS Programme of Research in South Africa, Nelson R Mandela School of Medicine, University of KwaZulu-Natal, Durban, South Africa; 3 Department of Epidemiology, Columbia University, New York, NY, USA; 4 Independent Science and Policy Consultant, San Francisco, CA, USA; 5 Leiden University, Leiden, The Netherlands; 6 Fundación Huesped, Buenos Aires, Argentina; 7 Department of Infectious Disease Epidemiology, Faculty of Epidemiology and Population Health, London School of Hygiene and Tropical Medicine, London, UK; 8 Department of Research, Makerere Medical School, Kampala, Uganda; 9 Social Policy Research Centre, University of New South Wales, Sydney, Australia; 10 Harvard School of Public Health, Boston, MA, USA; 11 Elizabeth Glaser Pediatric AIDS Foundation, Los Angeles, CA, USA; 12 European Association of Science Editors; Wiley-Blackwell, International House, London, UK; 13 Department of Research in Biomedicine and Health, University of Split School of Medicine, Split, Croatia; 14 Madison, WI, USA; 15 Division of AIDS, University of British Columbia, Vancouver, Canada; 16 BC Centre for Excellence in HIV/AIDS, St. Paul's Hospital, Providence Healthcare, Vancouver, Canada; 17 Portia Ltd, London, UK; 18 CIBERESP, University of Alicante, Alicante, Spain; 19 Department of Infection and Population Health, University College London, London, UK; 20 Department of Infectious Diseases, University of Dakar, Dakar, Senegal; 21 Jefferson Medical College of Thomas Jefferson University, Philadelphia, USA; 22 McGill University AIDS Centre, Jewish General Hospital, Montreal, QC, Canada

## Abstract

Sex and gender differences influence the health and wellbeing of men and women. Although studies have drawn attention to observed differences between women and men across diseases, remarkably little research has been pursued to systematically investigate these underlying sex differences. Women continue to be underrepresented in clinical trials, and even in studies in which both men and women participate, systematic analysis of data to identify potential sex-based differences is lacking. Standards for reporting of clinical trials have been established to ensure provision of complete, transparent and critical information. An important step in addressing the gender imbalance would be inclusion of a gender perspective in the next Consolidated Standards of Reporting Trials (CONSORT) guideline revision. Uniform Requirements for Manuscripts Submitted to Biomedical Journals, as a set of well-recognized and widely used guidelines for authors and biomedical journals, should similarly emphasize the ethical obligation of authors to present data analyzed by gender as a matter of routine. Journal editors are also promoters of ethical research and adequate standards of reporting, and requirements for inclusion of gender analyses should be integrated into editorial policies as a matter of urgency.

## Main text

A person's sex is an important health determinant. In a variety of instances, it has been shown that physiological differences between the sexes influence health outcomes. Genetic differences translate into distinct anatomy, result in variations in hormone production, and affect immunological and inflammatory responses. Ultimately, these multiple factors influence the health and wellbeing of men and women, explaining the diverse demographics and epidemiology of diseases, as well as distinctive responses to illness. In addition, there is a complex interplay between innate physiological sex differences and socioeconomic and behavioural gender differences that further affect health outcomes.

Although studies have drawn attention to observed differences between women and men across diseases, few have been designed to specifically do so. Some of these patterns have been observed as early as during foetal development, where external influence is minimized. For example, female infants may be at a substantially higher risk of acquiring HIV *in utero *than male infants, even after adjusting for confounders such as mothers' viral load and birth weight [1], and male infants seem more likely to be infected during breast feeding [2].

Similarly, women and men have been shown to display disparate immunity and immunopathology in response to a series of microbial infections, indicating a complex interaction between microbe-specific and gender-specific immune responses [3]. Autoimmune diseases, such as Systemic Lupus Erythematosus and rheumatoid arthritis, are more prevalent in women than men, and women more frequently reject allogeneic grafts after cardiac transplantation, with higher post-operative mortality [4]. A set of gender differences has also been observed in chronic pain, with some conditions further influenced differentially by age [5].

Remarkably little research has been pursued to systematically investigate these underlying sex differences. For too long, medical research has turned a blind eye to differences in disease prevalence, progression and clinical outcomes between women and men. Women continue to be underrepresented in clinical trials and are subject to medical practices based on data from a predominantly Caucasian male population [6]. Some would even argue that medicine as it is practiced today is less evidence-based for women than it is for men.

Even in studies in which both men and women participate, systematic analysis of data to identify potential sex-based differences is lacking. Similarly, editorial policies in scientific journals requiring or encouraging gender analysis in reported research are more often the exception than the rule.

Standards for reporting of clinical trials have been established to ensure provision of complete and transparent critical information. The Consolidated Standards of Reporting Trials (CONSORT) group came together in 1996 to provide uniform guidelines for accurate and adequate reporting of randomized clinical trials. Despite two revisions of the guidelines since its conception, the CONSORT statement fails to make any mention of sex and gender. Since the 2010 statement indicates that an aspiration of the group is to help improve design, conduct and reporting of trials, an important step in addressing the gender imbalance would be inclusion of a gender perspective in the next CONSORT guideline revision [7,8]. Others including UNAIDS have also requested such an inclusion in the CONSORT statement revision [8].

The International Committee of Medical Journal Editors (ICMJE) defines standards for scientific reporting in its Uniform Requirements for Manuscripts Submitted to Biomedical Journals [9]. Fortunately, these do propose analyses of data by such variables as age and sex *"where scientifically appropriate"*; this is an encouraging suggestion as the Uniform Requirements are a well-recognized and widely used set of guidelines for authors and biomedical journals. However, systematic provision of data disaggregated by sex (and age) is not only desirable, but should also be strongly recommended. ICMJE is certainly in a position to emphasize the ethical obligation of authors to present data analyzed by sex as a matter of routine.

Lately, there have been some positive developments on gender inclusion in medical research, and a growing number of efforts recognize the gender gap in science. A recent European gender summit co-organized by GenSET network and the European Science Foundation and supported by the European Commission may be one of the most comprehensive gatherings to review gender inequality and its consequences for science to date [10,11]. Presentations at the meeting highlighted how gender inequity becomes more pronounced as one ascends the academic career ladder, and how research falls short of ensuring that findings are relevant for subpopulations and will benefit both women and men. Data were also presented on how gender diversification positively stimulates innovation and productivity. The outcome of the summit was a manifesto signed by, among others, several science editors. The manifesto underlines that "the assertion that science is gender neutral is not the case", and calls for researchers, funding bodies, reviewers and journal editors to consider gender in research design, conduct and reporting [12].

*The Lancet *took a positive step following the gender summit in November 2011, when its editors introduced a very welcome policy, encouraging inclusion of sex and gender analysis in submitted manuscripts [13]. The *Journal of the International AIDS Society *also has a policy to this effect [14]. Our aspiration is that a similar policy be adopted across all scientific journals. Most importantly, however, we call on the ICMJE and CONSORT groups to recognize the importance of systematic analysis and reporting of sex differences from trials and to ensure that their guidelines introduce gender dimensions in their checklists and flowcharts, and recommend inclusion of this parameter as a matter of routine in the reporting of clinical data on human subjects.

Editors are often referred to as the gatekeepers of science, responsible for ensuring that what is published in scientific journals qualifies, both scientifically and ethically, as contributions to the collective pool of knowledge. Editors are also promoters of ethical research and adequate standards of reporting. Requirements for inclusion of gender analyses should not go unnoticed.

## Competing interests

SH is an employee of the International AIDS Society, and her salary is provided partly by unrestricted educational grants from the following pharmaceutical companies: Abbott, Boehringer Ingelheim, Gilead, Merck, Pfizer, Roche, Tibotec and ViiV Healthcare. PC has served as: Advisory Board Member at Avexa, Gilead, GSK, Myriad, Merck, Pfizer, Pharmasset, Schering Plough and Tibotec; Investigator at Avexa, Boehringer Ingelheim, Gilead, GSK, Roche, Merck, Pfizer, Pharmasset, Schering Plough, Tibotec, Abbott and BMS; Speaker (content and design performed by the speaker, no company control) for Abbott, BMS, Boehringer Ingelheim, GSK, Merck, Pfizer and Tibotec; and Scientific Advisor for Merck Sharp & Dohme, Pfizer, GSK, Avexa and Tibotec. He is not a shareholder in any pharmaceutical company, nor has he any commercial interest or investment in any pharmaceutical company. RM has served as an advisor to the BMS Foundation and the Merck Company Foundation.

JM is supported by the British Columbia Ministry of Health, through an Avant-Garde Award (No. 1DP1DA026182-01) from the National Institute of Drug Abuse (NIDA), at the US National Institutes of Health, and through a KT Award from the Canadian Institutes of Health Research. He has also received financial support from the International AIDS Society, United Nations AIDS Program, World Health Organization, National Institutes of Health Research-Office of AIDS Research, National Institute of Allergy & Infectious Diseases, the United States President's Emergency Plan for AIDS Relief, the Bill & Melinda Gates Foundation, French National Agency for Research on AIDS & Viral Hepatitis, the Public Health Agency of Canada, the University of British Columbia, Simon Fraser University, Providence Health Care and Vancouver Coastal Health Authority. He has received grants from Abbott, Biolytical, Boehringer-Ingelheim, Bristol-Myers Squibb, Gilead Sciences, Janssen, Merck and ViiV Healthcare. QAK, JDA, SEB, MJC, CH, EK, SK, JM, AM, HMN, EP, MTR-C, LS, PSS, KS, MAW have no competing interests to declare.

The opinions expressed in this article are those of the authors and do not necessarily reflect those of their respective organizations.

## Authors' contributions

SH wrote the first draft of the article. All other authors contributed equally to the manuscript by providing comments on subsequent drafts. All authors have read and approved the final version of this manuscript.
